# Effect of Feeding Selenium-Fertilized Alfalfa Hay on Performance of Weaned Beef Calves

**DOI:** 10.1371/journal.pone.0058188

**Published:** 2013-03-11

**Authors:** Jean A. Hall, Gerd Bobe, Janice K. Hunter, William R. Vorachek, Whitney C. Stewart, Jorge A. Vanegas, Charles T. Estill, Wayne D. Mosher, Gene J. Pirelli

**Affiliations:** 1 Department of Biomedical Sciences, College of Veterinary Medicine, Oregon State University, Corvallis, Oregon, United States of America; 2 Department of Animal and Rangeland Sciences, College of Agricultural Sciences, Oregon State University, Corvallis, Oregon, United States of America; 3 Linus Pauling Institute, Oregon State University, Corvallis, Oregon, United States of America; 4 Department of Clinical Sciences, College of Veterinary Medicine, Oregon State University, Corvallis, Oregon, United States of America; INRA, France

## Abstract

Selenium (Se) is an essential micronutrient in cattle, and Se-deficiency can affect morbidity and mortality. Calves may have greater Se requirements during periods of stress, such as during the transitional period between weaning and movement to a feedlot. Previously, we showed that feeding Se-fertilized forage increases whole-blood (WB) Se concentrations in mature beef cows. Our current objective was to test whether feeding Se-fertilized forage increases WB-Se concentrations and performance in weaned beef calves. Recently weaned beef calves (n = 60) were blocked by body weight, randomly assigned to 4 groups, and fed an alfalfa hay based diet for 7 wk, which was harvested from fields fertilized with sodium-selenate at a rate of 0, 22.5, 45.0, or 89.9 g Se/ha. Blood samples were collected weekly and analyzed for WB-Se concentrations. Body weight and health status of calves were monitored during the 7-wk feeding trial. Increasing application rates of Se fertilizer resulted in increased alfalfa hay Se content for that cutting of alfalfa (0.07, 0.95, 1.55, 3.26 mg Se/kg dry matter for Se application rates of 0, 22.5, 45.0, or 89.9 g Se/ha, respectively). Feeding Se-fertilized alfalfa hay during the 7-wk preconditioning period increased WB-Se concentrations (*P*_Linear_<0.001) and body weights (*P*_Linear_ = 0.002) depending upon the Se-application rate. Based upon our results we suggest that soil-Se fertilization is a potential management tool to improve Se-status and performance in weaned calves in areas with low soil-Se concentrations.

## Introduction

Selenium (Se) is an essential micronutrient of cattle. Provision of adequate Se is important to prevent Se-responsive diseases in growing cattle such as nutritional myodegeneration and Se-responsive unthriftiness [1]. Many parts of the world, including Oregon, USA, are known to have soil conditions conducive to deficient forage-Se content, potentially leading to clinical signs of Se deficiency in livestock grazing or fed crops raised on them [2]. Soils are the major source of Se for plants and soil-Se exits in various forms including selenides, elemental Se, selenites, selenates and organic Se compounds [3]. Soil-Se content varies considerably depending upon geographic location. Low soil pH and high concentrations of sulfur and phosphorus from fertilization decrease Se availability for plants. Leaching from the topsoil in areas of high rainfall or irrigation also lowers forage-Se content. Plant species also differ in their ability to incorporate Se from soil. Most forage plants are categorized as non-Se accumulator plants.

The bioavailability of Se is not straightforward because of wide variation in Se content of foods (determined by a combination of geographical and environmental factors) and chemical forms in which Se may be absorbed and metabolized [4]. In general, organic forms are absorbed and retained more efficiently than inorganic forms [5], [6]. Selenium is normally present in the diet in organic forms, e.g., as selenomethionine (SeMet) or selenocysteine (SeCys) [7]. Inorganic Na-selenite and Na-selenate are present in the diet in very small amounts.

Although the essentiality of Se has been known for five decades, the most effective method of Se delivery to cattle for optimum performance is still being investigated. Several means of administering Se to deficient ruminants are available [8]. For example, there are a number of injectable preparations, which often include vitamin E. Selenium can also be added to feed, mineral, and protein supplements. Sustained-release boluses with a life of several months may be used. Because of their weight, these boluses stay in the rumen whereby they gradually release Se. Selenium supplemented by these methods is usually inorganic Na-selenite or Na-selenate. One limitation of supplementing with inorganic Se in salt or feed is the apparent short duration of Se storage in the animal [8], [9]. Other limitations to these methods of Se delivery include individual variation or sporadic intake, extra labor requirements, added expenses, and seasonal grazing practices that result in limited access to Se for extended periods of time. Therefore, animals may be Se deficient by the end of the grazing season.

Agronomic biofortification is defined as increasing the bioavailable concentrations of essential elements in edible portions of crop plants through the use of fertilizers. The potential for using Se-containing fertilizers to increase forage Se concentrations and, thus, dietary Se intake has been demonstrated in Finland, New Zealand, and Australia where it has proven to be both effective and safe [10]–[14]. The predominant chemical form of Se in Se-fortified grains and hays is SeMet [7]. We previously reported that Se-replete beef cows fed Se-fertilized forage for 6 wk had elevated WB-Se concentrations for 20 wk, which ensured adequate WB-Se while grazing forage on Se-deficient soils [15]. We have also shown that the FDA-approved supplementation rate for sheep (0.3 mg of Se/kg of diet as fed, which is equivalent to 0.7 mg of Se/d or 4.9 mg of Se/wk per sheep) for organic Se supplementation was equally effective as supranutritional rates of Na-selenite supplementation (14.7 and 24.5 mg of Se/wk) in increasing whole-blood (WB) Se concentrations, demonstrating the greater oral bioavailability of organic Se in sheep. In addition, short-term exposure to Se-fertilized forage results in whole-body Se status sufficient to maintain adequate WB-Se concentrations throughout grazing periods when there is limited access to Se supplements [16]. In another study Stewart et al. [17] showed that growth and survival was better in lambs from ewes receiving Se-yeast at 5 times the FDA-allowed supplementation rate compared to lambs from ewes receiving the FDA-allowed supplementation rate or no Se.

The transition period between weaning and movement to a feedlot is one of the most stressful times for beef calves. Because Se plays an important role in the immune response in cattle [18], calves may have greater Se requirements during the transitional period. Performance in weaned beef calves is enhanced if a preconditioning program is utilized before calves enter the feedlot. Several weeks in a preconditioning program are recommended to reduce the stress associated with weaning, dehorning, castration, and vaccination with the goal of reducing morbidity and mortality after arrival at the feedlot. Preconditioning also reduces the number of calves pulled to sick pens, and improves weight gain and feed efficiency of calves after arrival at a feedlot [19]–[21].

The objectives of this study were to evaluate WB-Se status and performance in weaned beef calves fed alfalfa hay fertilized with Se at increasing rates for 7 wk in a preconditioning program prior to entering the feedlot. We hypothesized that feeding weaned beef calves forage fertilized with increasing amounts of Na-selenate would improve both WB-Se status and growth rate.

## Materials and Methods

### Animal Ethics Statement and Study Design

The experimental protocol was reviewed and approved by the Oregon State University Animal Care and Use Committee (ACUP Number: 4051). This was a prospective clinical trial of 7-wk duration (August 29 through October 14, 2010) involving 60 weaned beef calves, primarily of Angus breeding. The calves ranged in age from 4.5 to 6 mo (166±2 d; mean ± SEM) and originated from the Oregon State University Beef Ranch, Corvallis, OR, USA. Body weights at weaning ranged from 181 to 310 kg (239±3.6 kg, mean ± SEM), and body condition scores ranged from 6 to 7 (1 to 9 scale). There were 27 heifers and 33 steer calves in the study.

Corvallis is located at an elevation of 72 m, midway in the Willamette Valley, 74 km east of the Oregon Coast, and 137 km south of Portland. Like the rest of the Willamette Valley, Corvallis falls within the Marine West Coast climate zone with some Mediterranean characteristics. Temperatures are mild year round, with warm, dry, sunny summers and mild, wet winters with persistent overcast skies. Spring and fall are also moist seasons with persistent cloudiness, and light rain falling for extended periods. Winter snow is rare, but occasionally does fall, usually in the form of heavy wet snow, ranging between a dusting to several cm that does not persist on the ground for more than a day. During the mid-winter months after extended periods of rain, thick persistent fogs can form, sometimes lasting the entire day. Rainfall total is surprisingly variable, ranging from an average of 168.7 cm per year in the far northwest hills to 110.9 cm per year at Oregon State University, which is located in the center of Corvallis. Typical distribution of precipitation includes about 50 percent of the annual total from December through February, lesser amounts in the spring and fall, and very little during summer. Rainfall tends to vary inversely with temperatures, with the cooler months being the wettest, and the warmer summer months being the driest. Because of its close proximity to the coast range, Corvallis can experience slightly cooler temperatures, particularly in the hills, compared with the rest of the Willamette Valley. Despite this, temperatures dropping below freezing are a rare event. Average monthly temperatures for September are 25.1°C (high) and 9.0°C (low).

Using a randomized complete block design, calves were blocked at the time of weaning by body weight (BW) and then assigned to one of 4 treatment groups of 15 calves each. Ear tags were used to identify the calves. All calves were put together in a large dry field and fed non Se-fortified grass hay for 4 d. Calves were then placed by treatment group into dry barn lots (11–15 m^2^/calf; concrete flooring in open lots that were strip cleaned once weekly; dirt flooring in loafing sheds with 5–6 m^2^/calf; concrete bunks with 64–97 cm of feeder space/calf; all measurements exceeded requirements [22]) with continuous access to water, feed bunks, and shelter. Calves were fed alfalfa hay once daily. The alfalfa hay was grown in fields fertilized with sodium-selenate at an application rate of 0, 22.5, 45.0, or 89.9 g Se/ha. Calves were transitioned to their respective alfalfa hay sources over a 10-d period. Alfalfa hay was fed as follows: 0.64 kg/head/d 1; 0.79 kg/head/d 2; 1.59 kg/head/d 3; 3.2 kg/head/d 4 to 7; 4.77 kg/head/d 8 to 11; and 6.4 kg/head/d 12. In addition, grass hay was offered for the first 10 d, and then discontinued. By the beginning of the third wk, calves were consuming on average 6.4 kg (as fed) of alfalfa hay per head, which was approximately 2.6% of their BW. A specified quantity of hay was offered each day and intake was uniform among groups. Hay was available all day, but by the next morning bunkers were empty and less than 5% of hay (visual estimate) was wasted on the ground.

In addition, calves were fed grain-based concentrate (0.23 kg as fed/head/d for 5 wk and then 0.46 kg as fed/head/d for 2 wk; **Table 1**) containing a coccidiostat (Rumensin® 80; Elanco Animal Health Co, Indianapolis, IN; 0.0195%) and added Se (0.200 mg/kg). Grain concentrate was offered once a day beginning the day calves were placed into the barn lots; grain was placed into the bunkers and consumed before hay was fed. The grain concentrate consisted of 34% steam flaked corn, USDA grade 2; 28% rolled barley; 19% wheat middlings; 10% dried distiller’s grains with solubles from an ethanol plant; and 4% dried distiller’s grains containing mainly wheat. The ration was formulated for growing beef calves in the 200 to 300 kg weight range to achieve a target average daily gain of 0.5 kg/d.

**Table 1:** Alfalfa hay and grain concentrate nutrient composition (dry matter basis).

Nutrient	Alfalfa Hay	GrainConcentrate
Dry matter, g/kg	906	944
Crude protein, g/kg	183	158
Acid detergent fiber, g/kg	351	84
Neutral detergent fiber, g/kg	406	148
Nonfiber carbohydrates, g/kg^1^	323	608
Fat, g/kg	10	34
Ash, g/kg	78	52
Calcium, g/kg	15.3	7.4
Phosphorus, g/kg	2.7	5.4
Magnesium, g/kg	4.5	3.1
Potassium, g/kg	14.9	7.5
Sodium, g/kg	1.5	3.1
Copper, mg/kg	12	13
Iron, mg/kg	357	115
Manganese, mg/kg	43	61
Zinc, mg/kg	22	67

1 Nonfiber carbohydrates calculated by difference.

Prior to this study, dams and calves had free-choice access to a mineral supplement containing 120 mg/kg Se from sodium-selenite. After weaning and during this study, all calves had free-choice access to the same type of mineral supplement, however Se was not added to the mixture. The mineral supplement (dry matter basis) was in loose granular format and contained 57.0 to 64.0 g/kg calcium; 30.0 g/kg phosphorus; 503 to 553 g/kg salt (NaCl); 50.0 g/kg magnesium; 50 mg/kg cobalt; 2,500 mg/kg copper; 200 mg/kg manganese; 200 mg/kg iodine; 6,500 mg/kg zinc (Wilbur-Ellis Company, Clackamas, OR). During the first 10 d, one bloat block containing 13 mg/kg Se (Bloat Guard® POL6.6 Pressed, SWEETLIX® Livestock Supplement System; Mankato, MN) was also offered to each group of calves. Routine farm management practices, including vaccinations and deworming, were the same for all treatment groups with the exception that one calf in the 45.0 g Se/ha group was castrated at the beginning of the trial.

### Selenium Fortified-Alfalfa Hay and Other Selenium Analyses

The soil was enriched with Se by mixing sodium-selenate (RETORTE Ulrich Scharrer GmbH, Röthenbach, Germany) with water and spraying it onto the soil surface of an alfalfa field at an application rate of 0, 22.5, 45.0, or 89.9 g Se/ha immediately after the first cutting of hay in June 2010. Fields were approximately 1.2 ha each. The application rates were chosen based on work with Selcote Ultra® (10 g Se/kg as 1∶3 Na_2_SeO_4_:BaSeO_4_; Terralink, Vancouver, British Columbia, Canada) in previous studies [15], [16]. Second-cutting alfalfa hay was harvested 40 d after Se application and then analyzed for nutrient and Se content. Alfalfa yield was approximately 4.9 ton/ha. To determine whether Se remained in the soil after harvesting second-cutting alfalfa hay, third-cutting alfalfa hay was also harvested from the respective field plots after another 50 d and analyzed for Se content. A Penn State forage sampler was used to take 25 cores from random bales in each alfalfa hay source (0, 22.5, 45.0, or 89.9 g Se/ha). This sampling regime was repeated 3 times (all samples collected mid way through the feeding trial) for each alfalfa hay source. Core samples were mixed well and representative samples selected for analysis. Alfalfa hay samples were submitted to commercial laboratories for routine nutrient analysis (**Table 1**; Cumberland Analytical Services, Maugansville, MD) and Se analysis (Utah Veterinary Diagnostic Laboratory, Logan, UT). Alfalfa hay dry matter determination was completed at a temperature of 105°C for 12 to 14 h in a forced draught oven. Methods for crude protein (CP), acid detergent fiber (ADF), ash, and minerals were performed according to the Association of Official Analytical Chemists [23]. The neutral detergent fiber (NDF) was determined according to Van Soest et al. [24]. Soluble protein was determined according to Krishnamoorthy et al. [25]. Plant samples were prepared for Se analysis as previously described [26], and Se was analyzed using inductively coupled argon plasma emission spectroscopy (ICP-MS; ELAN 6000, Perkin Elmer, Shelton, CT). Quantification of Se was performed by the standard addition method, using a 4-point standard curve. A quality-control sample (in similar matrix) was analyzed after every 5 samples, and analysis was considered acceptable if the Se concentration of the quality-control sample fell within ±5% of the standard/reference value for the quality control.

Grain samples were prepared for Se analysis the same manner as alfalfa hay. Salt samples were ground using a mortar and pestle. The ground salt material (0.50 g) was placed into a labeled 30-ml digestion tube (Oak Ridge Teflon digestion tube, Nalge Nunc International, Rochester, NY). Trace metal-grade nitric acid (4.0 mL; Thermo Fisher Scientific Inc., Waltham, MA) was added to the digestion tubes. The tubes were then heated at 90°C for 1 h with the caps loose on the tubes. After digestion, tubes were allowed to cool and 5.0 mL of ultrapure water was added and the samples were again digested at 90°C for 1 h. Contents were increased to 10 mL by adding trace metal-grade nitric acid. One milliliter of the digest was transferred into another trace metal-free tube containing 9.0 mL of ultrapure water to make up a 5% (v/v) nitric acid matrix and was centrifuged at 520×g for 10 min. The supernatant was removed and the samples were analyzed to quantify Se using ICP-MS in the same manner as for plant and grain samples.

### Performance and Whole-blood Selenium Assay

Health was monitored daily during the 7-wk feeding trial. Body weights were measured at the beginning of the treatment period (baseline), and at 3 wk, 6 wk, and 7 wk (end of the Se supplementation period). To assess the effect of Se supplementation on WB-Se status, all calves were bled at 0 time (baseline) and once each week for 7 wk until study termination. Jugular venous blood was collected into evacuated ethylenediaminetetraacetic acid (EDTA) tubes (2 mL; final EDTA concentration 2 g/L; Becton Dickinson, Franklin Lakes, NJ) and stored on ice until they were frozen at −20°C. Whole-blood Se concentrations were determined by a commercial laboratory (Center for Nutrition, Diagnostic Center for Population and Animal Health, Michigan State University, E. Lansing, MI) using an ICP-MS method with modifications as previously described [15].

### Statistical Analyses

Statistical analyses were performed using SAS version 9.2 [27]. Whole-blood Se concentrations and BW were analyzed as repeated-measures-in-time using PROC MIXED. Fixed effects in the model were Se application rate (0, 22.5, 45.0, and 89.9 g Se/ha), sex of calf, (male, female), BW block, time (wk 0, 1, 2, 3, 4, 5, 6, and 7 of feeding experiment for WB-Se concentrations and wk 0, 3, 6, and 7 of feeding experiment for BW), and the interaction between Se application rate and time. Fixed effects in the model for BW were Se application rate, sex of calf, BW block, time (wk 0, 3, 6, and 7 of feeding experiment), and the interaction between Se application rate and time. An unstructured variance-covariance matrix was used to account for variation of measures within calves. The unstructured variance-covariance matrix provided the most parsimonious variance-covariance matrix based on the lowest value by the Aikaike Information Criterion.To evaluate the effect of Se application rate, linear, quadratic, and cubic contrasts were constructed. In addition, the linear response of the dependent variables Se forage content or WB-Se concentrations of beef calves to the independent variable Se fertilization rate were evaluated using univariate regression in PROC REG. Data are reported as least square means ± SEM. Statistical significance was declared at *P*≤0.05 and a tendency at 0.05<*P*≤0.10.

## Results

### Effect of Soil-Se Fertilization on Se Concentrations in Alfalfa Hay

**Figure 1:**
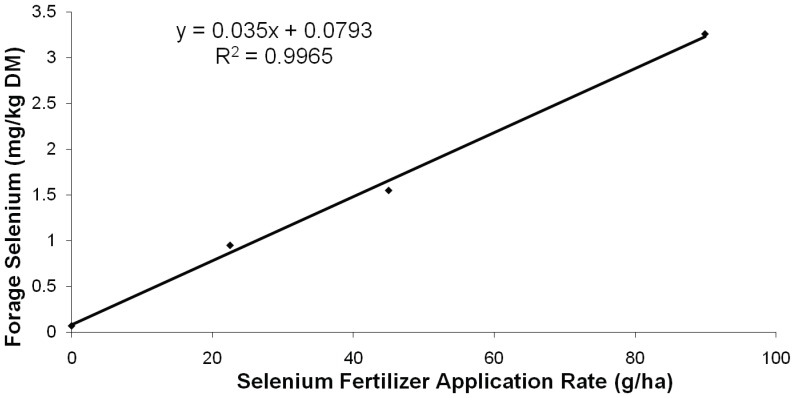
Relationship between amount of Se applied by fertilization (g Se/ha) and observed forage Se content (g Se/kg DM).

Fertilizing fields with increasing amounts of sodium-selenate increased in a dose-dependent manner the Se-content of second-cutting alfalfa hay from 0.07 to 0.95, 1.55, and 3.26 mg Se/kg dry matter for sodium-selenate application rates of 0 (non-fertilized control), 22.5, 45.0, or 89.9 g Se/ha, respectively (**Figure 1**). Subsequent third-cutting alfalfa hay had carry-over Se concentrations of 0.16, 0.28, and 0.60 mg Se/kg dry matter for sodium-selenate application rates of 22.5, 45.0, or 89.9 g Se/ha, which is equivalent to 16.8%, 18.1%, and 18.4% carry over, respectively.

### Effect of Supranutritional Se-supplementation to Se-replete Weaned Beef Calves on Whole- blood Se Concentrations and Performance during the 7-wk Feeding Period

Based on the total amount of alfalfa hay and grain concentrate fed to each group of calves, average dry matter intake per head was calculated at 5.59 kg/head/d for alfalfa hay starting on day 12, and 0.20 kg/head/d for grain concentrate for the first 5 wk and 0.40 kg/head/d for the last 2 wk. Using measured alfalfa hay and grain concentrate values for CP, net energy for gain (NE_g_), net energy for maintenance (NE_m_), and total digestible nutrients (TDN), we calculated CP (1.09 kg/head/d), NE_g_ (4.83 Mcal/head/d), NE_m_ (8.29 Mcal/head/d), and TDN consumption (3.7 kg/head/d) and compared them to National Research Council (NRC) [28] requirements {CP (0.44 kg/head/d), NE_g_ (0.62 Mcal/head/d), NE_m_ (4.50 Mcal/head/d), and TDN (2.9 kg/head/d)} for growing beef calves in this weight range to verify adequate nutrient intake for growth.

Calculated Se intake from alfalfa hay was 0.4, 5.3, 8.7, and 18.2 mg Se/head/d for calves consuming hay with Se concentrations of 0.07, 0.95, 1.55, and 3.26 mg Se/kg dry matter. The measured Se concentration of the grain concentrate was 1.41 mg Se/kg dry matter. Calculated Se intake from grain concentrate was 0.28 mg Se/head/d (first 5 wk) and 0.56 mg Se/head/d (last 2 wk). The average intake of mineral supplement was 17.5 mg/head/d. The measured Se concentration of the mineral supplement without added Se was 0.10 mg Se/kg dry matter. Calculated Se intake from the mineral supplement was 0.002 mg Se/head/d. The average intake of bloat block was 120 g/head/d during the first 10 d. The reported Se concentration of the bloat block offered during the first 10 d was 13 mg Se/kg. Calculated Se intake from the bloat block fed during the first 10 d was 1.55 mg Se/head/d.

**Figure 2:**
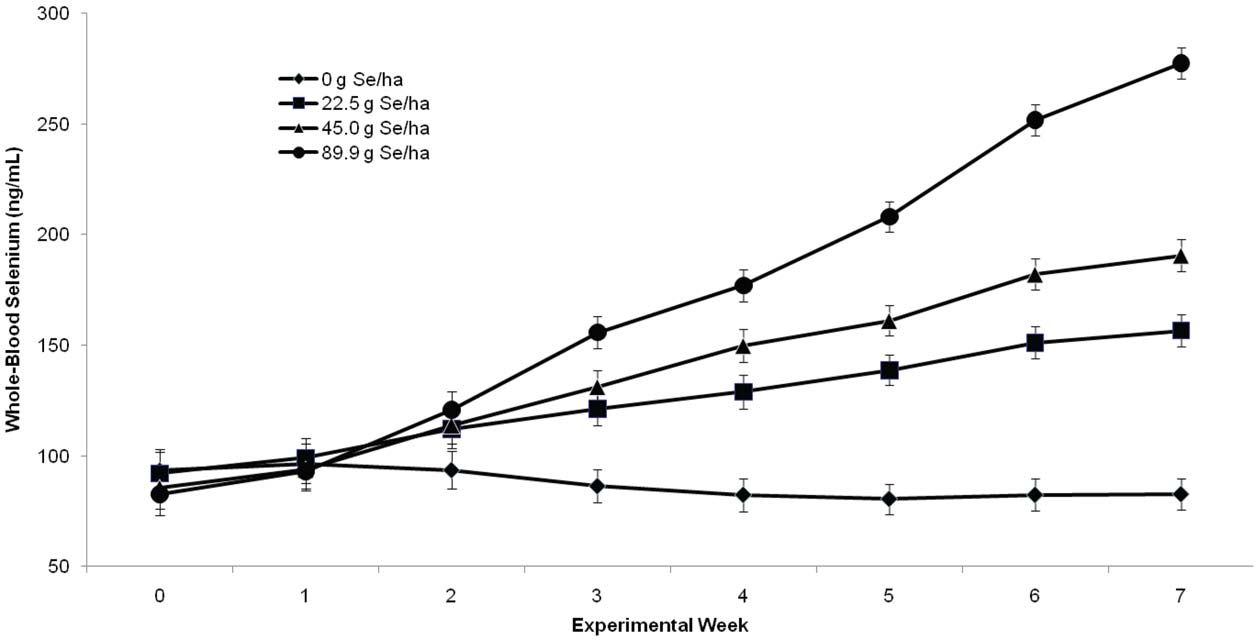
Comparison of whole-blood Se concentrations (mean ± SEM) in weaned beef calves consuming alfalfa hay grown in fields not fertilized with Se (0 g Se/ha), or harvested from fields fertilized with sodium-selenate at an application rate of 22.5, 45.0, or 89.9 g Se/ha for 7 wk (n = 15 calves per group). The normal reference interval for whole-blood Se concentrations of beef cattle is 120 to 300 ng/mL.

**Figure 3:**
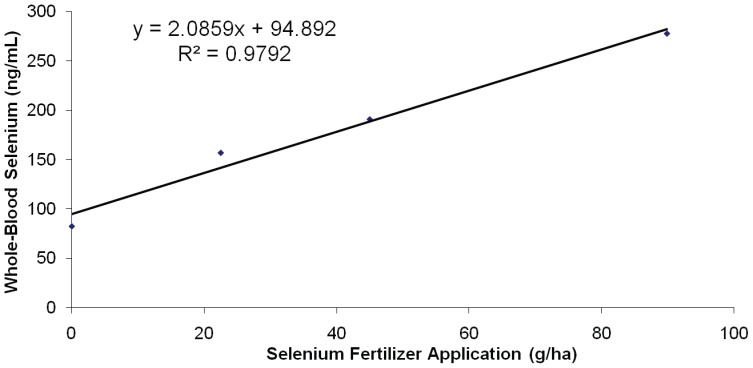
Relationship between amount of Se applied by fertilization (g Se/ha) and observed WB-Se content (ng/mL) in weaned beef calves consuming alfalfa hay grown in fields not fertilized with Se (0 g Se/ha), or harvested from fields fertilized with sodium-selenate at an application rate of 22.5, 45.0, or 89.9 g Se/ha for 7 wk (n = 15 calves per group). The normal reference interval for whole-blood Se concentrations of beef cattle is 120 to 300 ng/mL.

Feeding Se-fertilized alfalfa hay was effective at increasing WB-Se concentrations in weaned beef calves (*P*_Treatment_, *P*_Time,_ and *P*_Treatment_ ×_ Time_: *all P*<0.001; **Figure 2**). The normal reference interval for WB-Se concentrations of adult cows is 120–300 ng/mL [15]. Heifer calves had greater WB-Se concentrations than male calves (*P = *0.03). The WB-Se response increased with greater amounts of sodium-selenate applied to the soil (*P*_Linear_<0.001; **Figure 3**). No significant quadratic (*P = *0.11) or cubic (*P = *0.30) Se-dose response was detected. The WB-Se concentrations continued to increase throughout the 7-wk feeding period.

**Figure 4:**
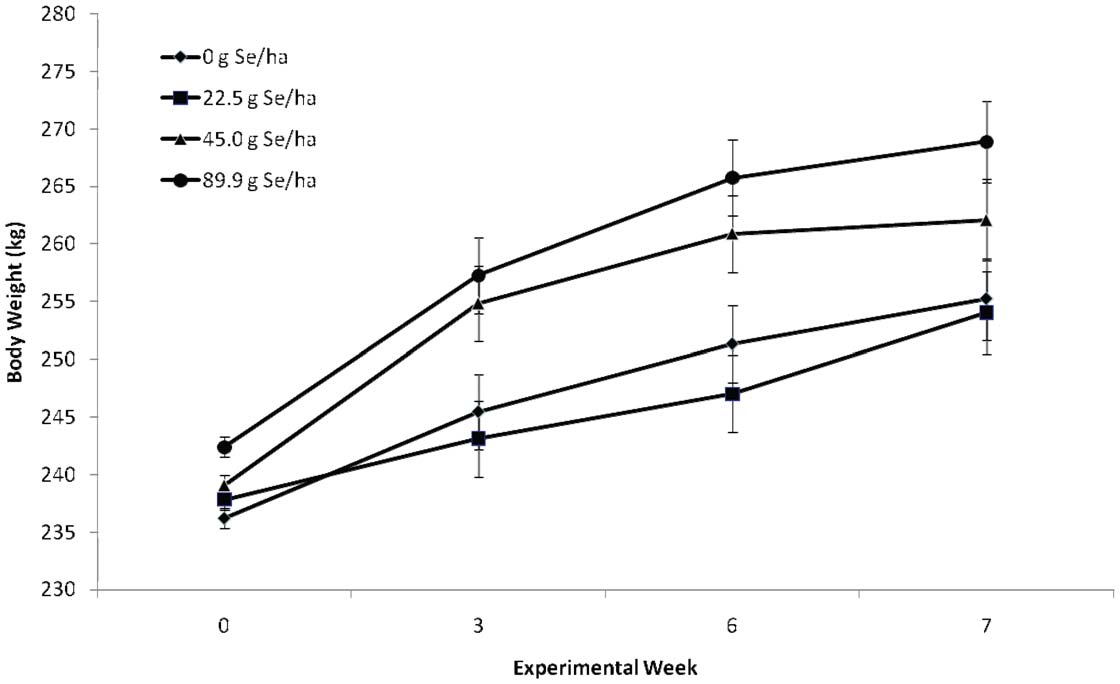
Comparison of block-adjusted BW (kg; mean ± SEM) of weaned beef calves (primarily of Angus breeding and ranging in age from 4.5-to-6-mo) after consuming alfalfa hay grown in fields not fertilized with Se (0 g Se/ha), or harvested from fields fertilized with sodium-selenate at an application rate of 22.5, 45.0, or 89.9 g Se/ha for 7 wk (n = 15 calves per group). Initial BW (baseline) ranged from 181 to 310 kg (239±3.6 kg, mean ± SEM). Final BW (7-wk) ranged from 183 to 346 kg (260±4.1 kg, mean ± SEM).

Feeding Se-fertilized alfalfa hay was effective at increasing BW in weaned beef calves (*P*_Treatment_ = 0.002; *P*_Time_<0.001; and *P*_Treatment_ ×_Time_ *= *0.03; **Figure 4**). Calf sex did not affect BW (*P = *0.98). The BW response increased with greater amounts of sodium-selenate applied to the soil (*P*_Linear_<0.001; **Figure 4**). No significant quadratic (*P = *0.84) or cubic (*P = *0.11) Se-dose response was detected. Weight data were not confounded by adverse health events in the preconditioning period because only two calves were retreated for pink eye during the first week of the feeding period (existent before the start of the experiment). None of the calves died during the 7-wk feeding period or showed signs of Se-deficiency or Se-toxicosis.

## Discussion

The objectives of this study were to evaluate whether fertilizing the soil of alfalfa hay fields with increasing amounts of sodium-selenate, and subsequent feeding of Se-fertilized alfalfa hay to recently weaned beef calves would improve in a dose-dependent manner WB-Se status and, consequently, increase growth rate in the preconditioning period prior to entering the feedlot. Fertilizing alfalfa hay fields with sodium-selenate increased Se content of alfalfa hay in a dose-dependent manner. Feeding Se-fertilized alfalfa hay during the 7-wk preconditioning program increased WB-Se concentrations and BW in Se-replete calves in a dose-dependent manner. Our results suggest that fertilization of alfalfa fields with sodium-selenate is a potential management tool to improve Se status and performance in weaned beef calves.

### Effect of Fertilizing Soil with Increasing Application Rates of Sodium-selenate on Se Concentrations in Alfalfa Hay

Agronomic Se-biofortification has been used in several countries with regions of low soil-Se concentrations including Finland, Denmark, New Zealand, and the United Kingdom to increase Se concentrations in the food chain [10], [29], [30]. In the United States, the use of feedstuffs that are naturally high in Se content is not regulated; Se fertilization, however, is not allowed in any state except for Oregon, where the Department of Agriculture does not control the use of Se as a plant fertilizer. Therefore, in Oregon it is possible to produce feedstuffs with increased Se concentrations by applying Se as a fertilizer. Plants absorb Se from the soil in the form of selenate and synthesize selenoamino acids with SeMet being the major selenocompound in grassland legumes [7].

We have shown that sodium-selenate can be solubilized in water and sprayed onto soil surfaces of established alfalfa hay fields after the first cutting of alfalfa hay at three application rates in a 1×, 2×, and 4× ratio (0, 22.5, 45.0, or 89.9 g Se/ha). Hay harvested from respective field plots has a similar dose-dependent Se content (0.07, 0.95, 1.55, and 3.26 mg Se/kg dry matter) (**Figure 1**).The Se fertilizer application rates in the current study were chosen based upon WB-Se concentrations attained and absence of clinical signs of toxicosis in previous Se-fertilization studies [15], [16].

In Hall et al. [15], Selcote Ultra® (10 g Se/kg as sodium selenate; Terralink, Vancouver, British Columbia, Canada) was mixed with urea-sol fertilizer and applied to pasture at a rate of 3.4 kg Selcote/ha (34 g Se/ha; 1.5× our lowest Se application rate in the current study). This resulted in forage Se of 0.11, 1.52, and 1.06 mg/kg (dry matter basis) at pre-fertilization, day 1, and day 42 of the grazing period, respectively. Beef cows grazing this forage had WB-Se concentrations of 186±5 ng/mL immediately post-grazing (day 42), which was within the normal reference interval for WB-Se concentrations of adult cows at the Michigan State University diagnostic laboratory (120–300 ng/mL). In a sheep study [16], Se applied to subclover-fescue sward type pasture at 3.4 kg Selcote/ha resulted in Se concentrations of up to 2.02 mg/kg (dry matter basis) in green forage grazed by the sheep, and WB-Se concentrations in ewes after grazing for 6 weeks of 573±20 ng/mL, with no clinical signs of Se toxicosis. When comparing the results of the current study with results from these two studies and one other that was conducted by our group that used Selcote Ultra® for Se fertilization (all at different locations in Oregon), a linear relationship between sodium-selenate application rate and Se content of forage was observed (forage selenium concencentrations in mg/kg DM = 0.0299×selenium fertilizer application rate in g/ha +0.3297; r^2^ = 0.9967**)** [15], [16].

The linear relationship between Se fertilizer application rate and forage Se concentration is surprising given the fact that plant species, field location, and soil differed among the studies [15], [16]. It is well documented that plant species, chemical species of Se, and soil pH, sulfur, and iron content alter Se availability for plant uptake [31], [32]. Based upon our results, we suggest that Se content of soil primarily determines Se content of common forage species. This is supported by the observation that, regardless of initial Se-application rate, subsequent third-cutting alfalfa hay had approximately 18% of the Se content of hay harvested after initial application of Se. The Se concentrations of third-cutting alfalfa hay were 0.16, 0.28, and 0.60 mg Se/kg (dry matter basis) for sodium-selenate application rates of 22.5, 45.0, or 89.9 g Se/ha, respectively. To our knowledge, this is the first report of Se carry-over in the soil to the next cutting of alfalfa hay.

### Effects of Feeding Alfalfa Hay Fertilized with Increasing Application Rates of Se for 7 wk on Whole-blood Se Concentrations and Performance of Weaned Beef Calves

Feeding Se-fertilized alfalfa hay was effective at increasing WB-Se concentrations in Se-replete weaned beef calves. The WB-Se concentrations increased with greater amounts of sodium-selenate applied to soil (**Figures 2** **and** **3**). For example, the increase in WB-Se concentrations for calves consuming alfalfa hay from fields fertilized with 89.9 vs. 45.0 g Se/ha was double the increase for calves consuming alfalfa hay from fields fertilized with 45.0 vs. 22.5 g Se/ha. Combining our results with a previous study in mature beef cows [15] indicates that there is a linear relationship between sodium-selenate application rate and WB-Se concentrations in beef cattle (WB-Se concentration in ng/mL = 1.9688×selenium fertilizer application rate in g/ha +105.67; r^2^ = 0.9594). The WB-Se concentrations continued to increase throughout the 7-wk feeding period.

The majority of dietary Se was supplied by the alfalfa hay, except in calves consuming non-Se fertilized alfalfa hay. In the first 5 wk, 0.28 mg Se/head/d was provided by the grain concentrate with essentially none provided by the mineral supplement. In the last 2 wk, 0.56 mg Se/head/d was provided by the grain concentrate. Thus, in the last 2 wk, total dietary Se intake was 0.96, 5.86, 9.26, and 18.76 mg Se/d for calves consuming alfalfa hay with sodium-selenite application rates of 0, 22.5, 45.0, or 89.9 g Se/ha (alfalfa hay provided 41, 90, 94, and 97% of dietary Se intake, respectively). During the first 10 d of the feeding trial, calculated Se intake from the bloat block was 1.55 mg Se/head/d. This represented the greatest source of Se for this short-term period in those calves receiving non-Se fertilized alfalfa hay.

In the United States, the FDA [33] allows Se to be added to cattle diets as sodium-selenite, sodium-selenate, or Se-yeast in complete feeds not to exceed 0.3 mg of Se/kg of diet (as fed basis), or in supplements for limit feeding not to exceed 3 mg of Se/head/day. The non-Se fortified alfalfa hay (0.07 mg Se/kg dry matter) was below this level. We were able to provide additional dietary Se using Se-fortified alfalfa hay. Our results for WB-Se concentrations in weaned beef calves fed Se-biofortified hay are similar to what we reported [15] when grazing adult beef cattle on Se-fertilized forage. No clinical signs of Se deficiency or toxicosis were observed.

Our results are consistent with SeMet from the grassland legumes being absorbed in the duodenum and incorporated into general body proteins in place of methionine. The concentration of SeMet is not regulated and ultimately reflects dietary intake [7], [32]. Selenomethionine acts as a storage form of Se in body proteins, including hemoglobin and albumin in WB, from which it is slowly released by protein catabolism. The SeMet can also be trans-selenated into selenocysteine and subsequently used to provide Se requirements for selenoprotein synthesis [34].

Production benefits of agronomic Se biofortification were assessed by comparing BW gains at the end of the 7-wk Se-supplementation period. Feeding Se-fertilized alfalfa hay increased BW in a linear manner (**Figure 4**). Calves receiving Se-fertilized alfalfa hay with the highest concentration of Se had the highest BW. There results were not confounded by morbidly because none of the calves were sick during the 7-wk preconditioning period. None experienced bloating, went off feed, or showed any clinical signs of illness. These results are consistent with our previous results for lambs [17] whereby ewes raising multiples that received the highest Se-yeast dose (24.5 mg Se/wk) had heavier lambs at weaning (120 d of age) in yr 1 of supplementation and at 60 d of age in yr 2 of supplementation than ewes receiving Se-yeast at the maximum FDA-allowed level (4.9 mg Se/wk).

Selenium’s role in animal performance is based upon the functions of selenoproteins, many of which have antioxidant activities [4]. Although reactive oxygen species and free radicals are a natural result of the body’s normal metabolic activity, excessive stress as a result of weaning, dietary and environmental changes, comingling with other animals, and disease can lead to the over production of free radicals or accumulation of free radicals because of a lack of antioxidants. Therefore, it is important that micronutrients involved in antioxidant functions be present in tissues to provide oxidant-antioxidant balance. Although the level of Se supplementation needed for adequate performance may be less under optimum conditions, in periods of transition such as weaning, preconditioning, and shipping, where stress is a confounding factor for optimum performance, Se requirements may be increased.

Because all calves were visually healthy, it is unlikely that the observed BW response to Se supplementation is explained solely by the antioxidant activity of selenoproteins. Two selenoprotein families, the iodothyronine deiodinases, responsible for metabolism of thyroid hormones, and the thioredoxin reductases, responsible for reducing thioredoxin, are directly or indirectly through regulation of transcription factors, involved in cell growth and control of apoptosis, as well as maintenance of cellular redox status [35]. Our results suggest that supranutritional Se supplementation may have growth-promoting properties in beef cattle. Future studies are warranted to examine the effects of supranutritional Se supplementation on gene and protein expression of the iodothyronine deiodinases, thioredoxin reductases, and transcription factors regulating cell growth.

In summary, Se fertilization of alfalfa fields in a region with Se deficient soils increased in a dose-dependent manner the Se content of alfalfa hay. Supranutritional Se supplementation of recently weaned beef calves with Se-fortified alfalfa hay resulted in increased WB-Se concentrations and improved growth rates. Our results suggest that building Se-body reserves by feeding supranutritional Se levels from sodium-selenate fertilized alfalfa hay during the preconditioning program is an effective management strategy to optimize growth and health in weaned beef calves.
